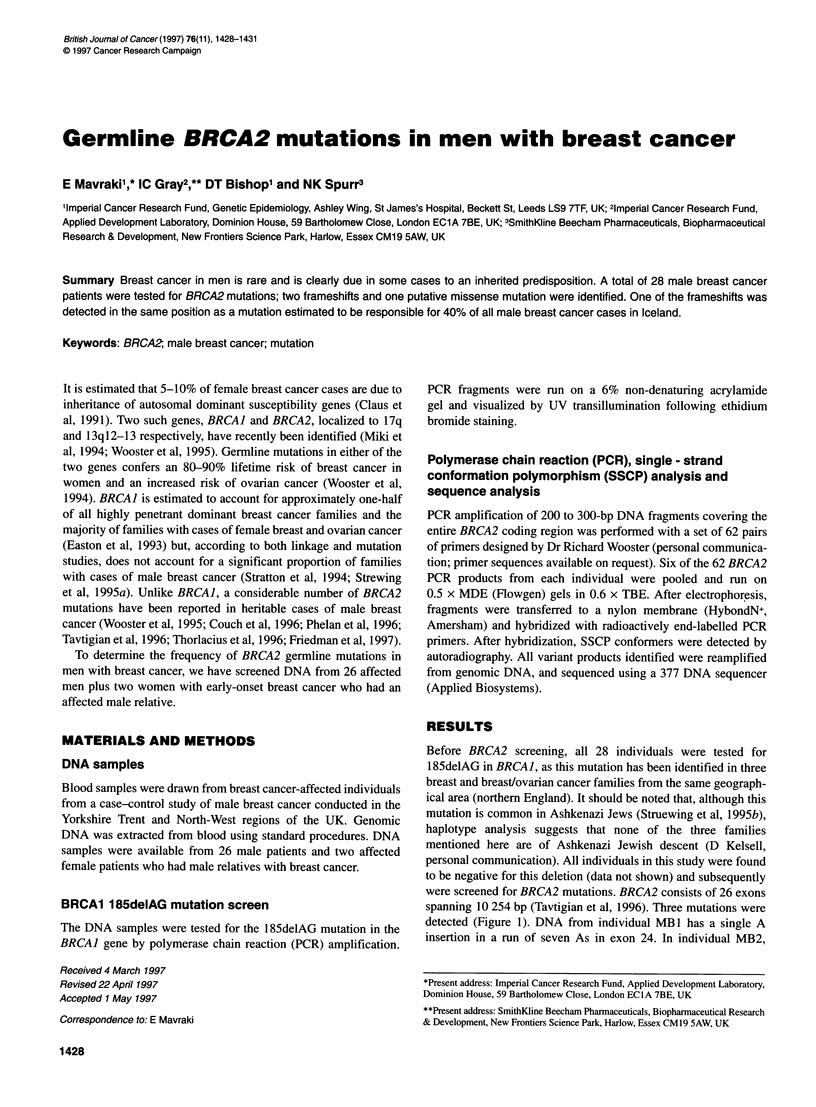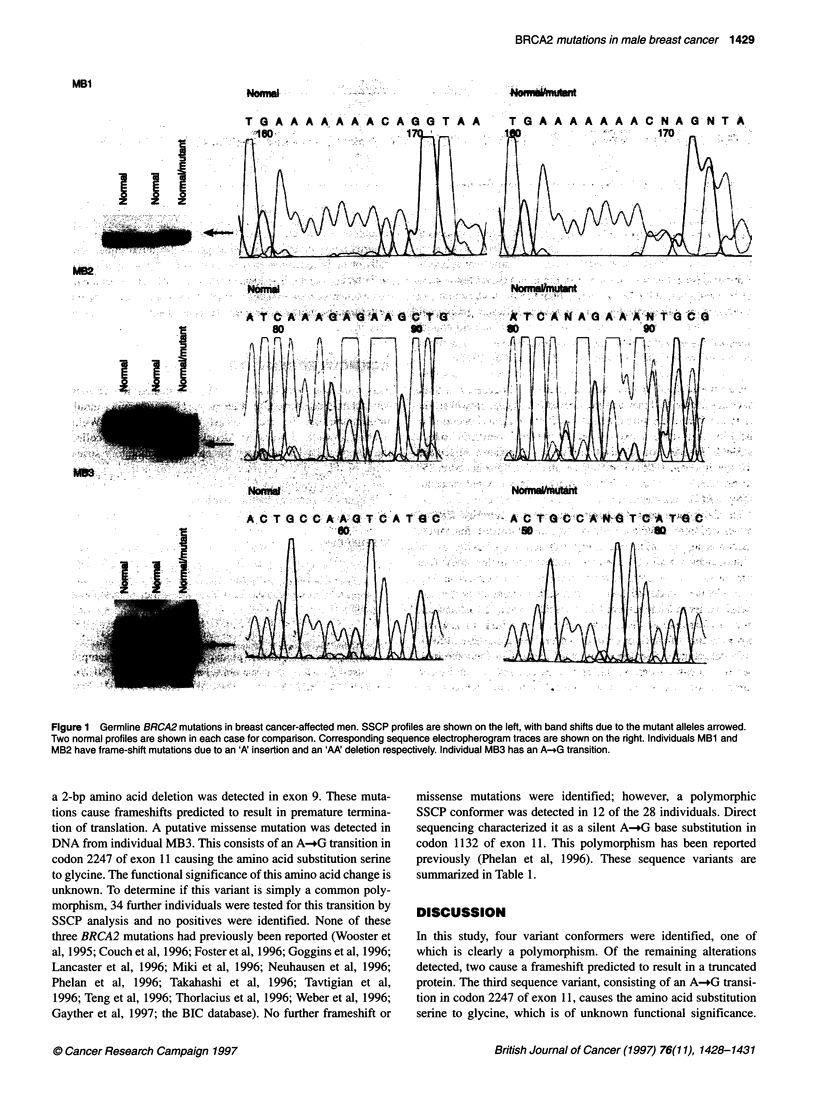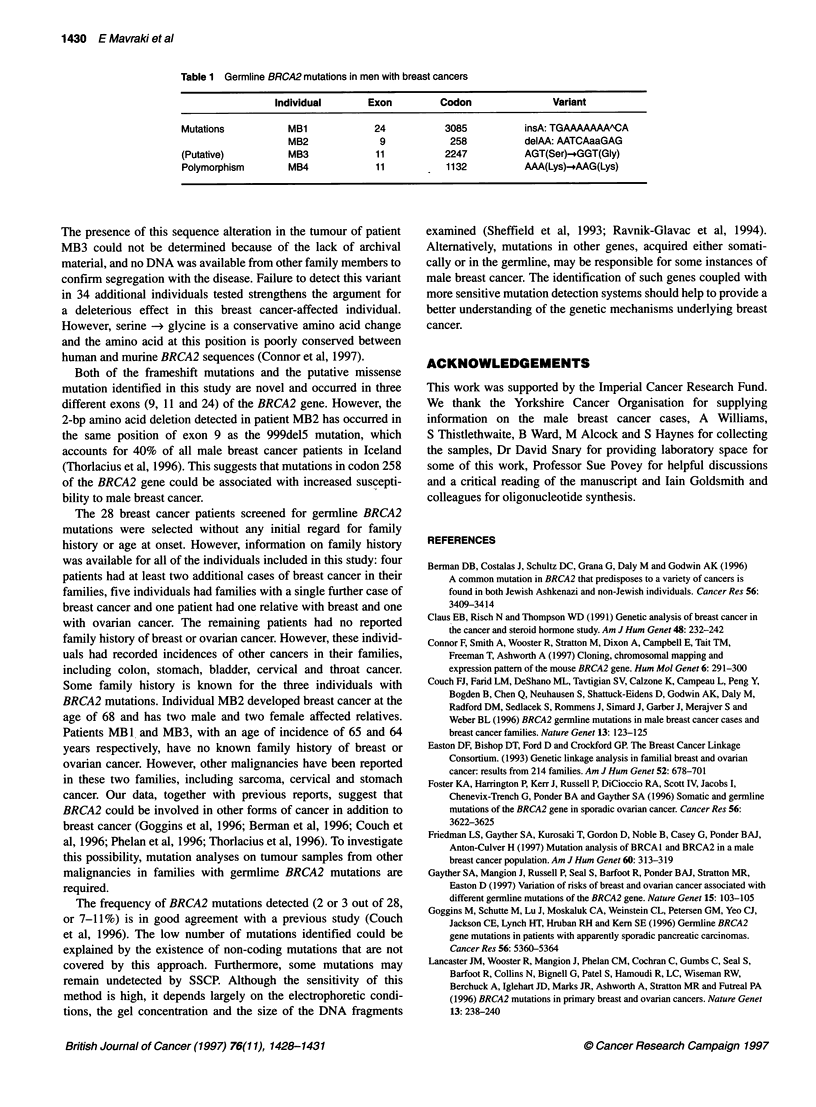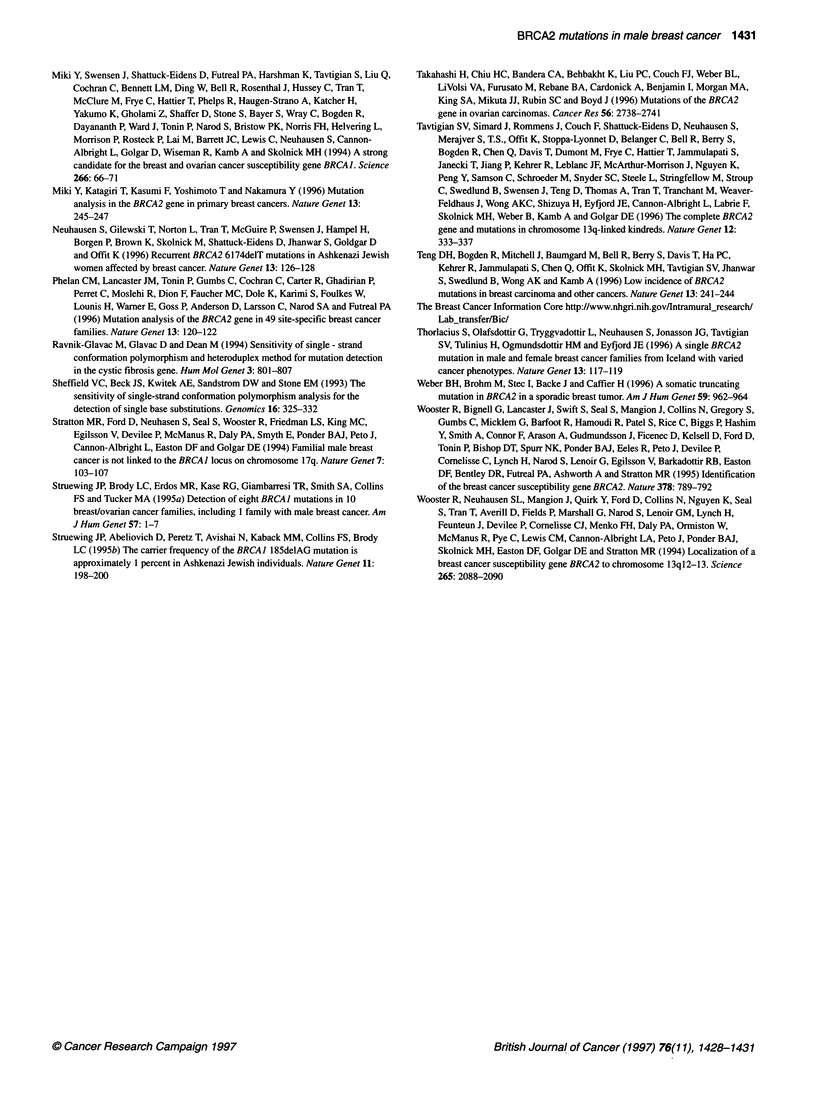# Germline BRCA2 mutations in men with breast cancer.

**DOI:** 10.1038/bjc.1997.574

**Published:** 1997

**Authors:** E. Mavraki, I. C. Gray, D. T. Bishop, N. K. Spurr

**Affiliations:** Imperial Cancer Research Fund, Genetic Epidemiology, St James's Hospital, Leeds, UK.

## Abstract

**Images:**


British Journal of Cancer (1997) 76(11), 1428–1431
© 1997 Cancer Research Campaign

### Germline *BRCA2* mutations in men with breast cancer

**E Mavraki[1],* IC Gray[2],** DT Bishop[1] and NK Spurr[3]**

[1]Imperial Cancer Research Fund, Genetic Epidemiology, Ashley Wing, St James's Hospital, Beckett St, Leeds LS9 7TF, UK; [2]Imperial Cancer Research Fund, Applied Development Laboratory, Dominion House, 59 Bartholomew Close, London EC1A 7BE, UK; [3]SmithKline Beecham Pharmaceuticals, Biopharmaceutical Research & Development, New Frontiers Science Park, Harlow, Essex CM19 5AW, UK

**Summary** Breast cancer in men is rare and is clearly due in some cases to an inherited predisposition. A total of 28 male breast cancer patients were tested for *BRCA2* mutations; two frameshifts and one putative missense mutation were identified. One of the frameshifts was detected in the same position as a mutation estimated to be responsible for 40% of all male breast cancer cases in Iceland.

**Keywords:** *BRCA2*; male breast cancer; mutation

It is estimated that 5–10% of female breast cancer cases are due to inheritance of autosomal dominant susceptibility genes (Claus et al, 1991). Two such genes, *BRCA1* and *BRCA2*, localized to 17q and 13q12–13 respectively, have recently been identified (Miki et al, 1994; Wooster et al, 1995). Germline mutations in either of the two genes confers an 80–90% lifetime risk of breast cancer in women and an increased risk of ovarian cancer (Wooster et al, 1994). *BRCA1* is estimated to account for approximately one-half of all highly penetrant dominant breast cancer families and the majority of families with cases of female breast and ovarian cancer (Easton et al, 1993) but, according to both linkage and mutation studies, does not account for a significant proportion of families with cases of male breast cancer (Stratton et al, 1994; Strewing et al, 1995*a*). Unlike *BRCA1*, a considerable number of *BRCA2* mutations have been reported in heritable cases of male breast cancer (Wooster et al, 1995; Couch et al, 1996; Phelan et al, 1996; Tavtigian et al, 1996; Thorlacius et al, 1996; Friedman et al, 1997).

To determine the frequency of *BRCA2* germline mutations in men with breast cancer, we have screened DNA from 26 affected men plus two women with early-onset breast cancer who had an affected male relative.

### MATERIALS AND METHODS

### DNA samples

Blood samples were drawn from breast cancer-affected individuals from a case–control study of male breast cancer conducted in the Yorkshire Trent and North-West regions of the UK. Genomic DNA was extracted from blood using standard procedures. DNA samples were available from 26 male patients and two affected female patients who had male relatives with breast cancer.

### BRCA1 185delAG mutation screen

The DNA samples were tested for the 185delAG mutation in the *BRCA1* gene by polymerase chain reaction (PCR) amplification. PCR fragments were run on a 6% non-denaturing acrylamide gel and visualized by UV transillumination following ethidium bromide staining.

### Polymerase chain reaction (PCR), single - strand conformation polymorphism (SSCP) analysis and sequence analysis

PCR amplification of 200 to 300-bp DNA fragments covering the entire *BRCA2* coding region was performed with a set of 62 pairs of primers designed by Dr Richard Wooster (personal communication; primer sequences available on request). Six of the 62 *BRCA2* PCR products from each individual were pooled and run on 0.5 × MDE (Flowgen) gels in 0.6 × TBE. After electrophoresis, fragments were transferred to a nylon membrane (HybondN+, Amersham) and hybridized with radioactively end-labelled PCR primers. After hybridization, SSCP conformers were detected by autoradiography. All variant products identified were reamplified from genomic DNA, and sequenced using a 377 DNA sequencer (Applied Biosystems).

### RESULTS

Before *BRCA2* screening, all 28 individuals were tested for 185delAG in *BRCA1*, as this mutation has been identified in three breast and breast/ovarian cancer families from the same geographical area (northern England). It should be noted that, although this mutation is common in Ashkenazi Jews (Struewing et al, 1995*b*), haplotype analysis suggests that none of the three families mentioned here are of Ashkenazi Jewish descent (D Kelsell, personal communication). All individuals in this study were found to be negative for this deletion (data not shown) and subsequently were screened for *BRCA2* mutations. *BRCA2* consists of 26 exons spanning 10 254 bp (Tavtigian et al, 1996). Three mutations were detected (Figure 1). DNA from individual MB1 has a single A insertion in a run of seven As in exon 24. In individual MB2,

*Received 4 March 1997*
*Revised 22 April 1997*
*Accepted 1 May 1997*

*Correspondence to:* E Mavraki

*Present address: Imperial Cancer Research Fund, Applied Development Laboratory, Dominion House, 59 Bartholomew Close, London EC1A 7BE, UK

**Present address: SmithKline Beecham Pharmaceuticals, Biopharmaceutical Research & Development, New Frontiers Science Park, Harlow, Essex CM19 5AW, UK

**Figure 1** Germline *BRCA2* mutations in breast cancer-affected men. SSCP profiles are shown on the left, with band shifts due to the mutant alleles arrowed. Two normal profiles are shown in each case for comparison. Corresponding sequence electropherogram traces are shown on the right. Individuals MB1 and MB2 have frame-shift mutations due to an 'A' insertion and an 'AA' deletion respectively. Individual MB3 has an A→G transition.

a 2-bp amino acid deletion was detected in exon 9. These mutations cause frameshifts predicted to result in premature termination of translation. A putative missense mutation was detected in DNA from individual MB3. This consists of an A→G transition in codon 2247 of exon 11 causing the amino acid substitution serine to glycine. The functional significance of this amino acid change is unknown. To determine if this variant is simply a common polymorphism, 34 further individuals were tested for this transition by SSCP analysis and no positives were identified. None of these three *BRCA2* mutations had previously been reported (Wooster et al, 1995; Couch et al, 1996; Foster et al, 1996; Goggins et al, 1996; Lancaster et al, 1996; Miki et al, 1996; Neuhausen et al, 1996; Phelan et al, 1996; Takahashi et al, 1996; Tavtigian et al, 1996; Teng et al, 1996; Thorlacius et al, 1996; Weber et al, 1996; Gayther et al, 1997; the BIC database). No further frameshift or missense mutations were identified; however, a polymorphic SSCP conformer was detected in 12 of the 28 individuals. Direct sequencing characterized it as a silent A→G base substitution in codon 1132 of exon 11. This polymorphism has been reported previously (Phelan et al, 1996). These sequence variants are summarized in Table 1.

### DISCUSSION

In this study, four variant conformers were identified, one of which is clearly a polymorphism. Of the remaining alterations detected, two cause a frameshift predicted to result in a truncated protein. The third sequence variant, consisting of an A→G transition in codon 2247 of exon 11, causes the amino acid substitution serine to glycine, which is of unknown functional significance.

**Table 1** Germline *BRCA2* mutations in men with breast cancers

| | Individual | Exon | Codon | Variant |
|---|---|---|---|---|
| Mutations | MB1 | 24 | 3085 | insA: TGAAAAAAA^CA |
| | MB2 | 9 | 258 | delAA: AATCAaaGAG |
| (Putative) | MB3 | 11 | 2247 | AGT(Ser)→GGT(Gly) |
| Polymorphism | MB4 | 11 | 1132 | AAA(Lys)→AAG(Lys) |

The presence of this sequence alteration in the tumour of patient MB3 could not be determined because of the lack of archival material, and no DNA was available from other family members to confirm segregation with the disease. Failure to detect this variant in 34 additional individuals tested strengthens the argument for a deleterious effect in this breast cancer-affected individual. However, serine → glycine is a conservative amino acid change and the amino acid at this position is poorly conserved between human and murine *BRCA2* sequences (Connor et al, 1997).

Both of the frameshift mutations and the putative missense mutation identified in this study are novel and occurred in three different exons (9, 11 and 24) of the *BRCA2* gene. However, the 2-bp amino acid deletion detected in patient MB2 has occurred in the same position of exon 9 as the 999del5 mutation, which accounts for 40% of all male breast cancer patients in Iceland (Thorlacius et al, 1996). This suggests that mutations in codon 258 of the *BRCA2* gene could be associated with increased susceptibility to male breast cancer.

The 28 breast cancer patients screened for germline *BRCA2* mutations were selected without any initial regard for family history or age at onset. However, information on family history was available for all of the individuals included in this study: four patients had at least two additional cases of breast cancer in their families, five individuals had families with a single further case of breast cancer and one patient had one relative with breast and one with ovarian cancer. The remaining patients had no reported family history of breast or ovarian cancer. However, these individuals had recorded incidences of other cancers in their families, including colon, stomach, bladder, cervical and throat cancer. Some family history is known for the three individuals with *BRCA2* mutations. Individual MB2 developed breast cancer at the age of 68 and has two male and two female affected relatives. Patients MB1 and MB3, with an age of incidence of 65 and 64 years respectively, have no known family history of breast or ovarian cancer. However, other malignancies have been reported in these two families, including sarcoma, cervical and stomach cancer. Our data, together with previous reports, suggest that *BRCA2* could be involved in other forms of cancer in addition to breast cancer (Goggins et al, 1996; Berman et al, 1996; Couch et al, 1996; Phelan et al, 1996; Thorlacius et al, 1996). To investigate this possibility, mutation analyses on tumour samples from other malignancies in families with germlime *BRCA2* mutations are required.

The frequency of *BRCA2* mutations detected (2 or 3 out of 28, or 7–11%) is in good agreement with a previous study (Couch et al, 1996). The low number of mutations identified could be explained by the existence of non-coding mutations that are not covered by this approach. Furthermore, some mutations may remain undetected by SSCP. Although the sensitivity of this method is high, it depends largely on the electrophoretic conditions, the gel concentration and the size of the DNA fragments examined (Sheffield et al, 1993; Ravnik-Glavac et al, 1994). Alternatively, mutations in other genes, acquired either somatically or in the germline, may be responsible for some instances of male breast cancer. The identification of such genes coupled with more sensitive mutation detection systems should help to provide a better understanding of the genetic mechanisms underlying breast cancer.

### ACKNOWLEDGEMENTS

This work was supported by the Imperial Cancer Research Fund. We thank the Yorkshire Cancer Organisation for supplying information on the male breast cancer cases, A Williams, S Thistlethwaite, B Ward, M Alcock and S Haynes for collecting the samples, Dr David Snary for providing laboratory space for some of this work, Professor Sue Povey for helpful discussions and a critical reading of the manuscript and Iain Goldsmith and colleagues for oligonucleotide synthesis.

### REFERENCES

Berman DB, Costalas J, Schultz DC, Grana G, Daly M and Godwin AK (1996) A common mutation in *BRCA2* that predisposes to a variety of cancers is found in both Jewish Ashkenazi and non-Jewish individuals. *Cancer Res* **56**: 3409–3414

Claus EB, Risch N and Thompson WD (1991) Genetic analysis of breast cancer in the cancer and steroid hormone study. *Am J Hum Genet* **48**: 232–242

Connor F, Smith A, Wooster R, Stratton M, Dixon A, Campbell E, Tait TM, Freeman T, Ashworth A (1997) Cloning, chromosomal mapping and expression pattern of the mouse *BRCA2* gene. *Hum Mol Genet* **6**: 291–300

Couch FJ, Farid LM, DeShano ML, Tavtigian SV, Calzone K, Campeau L, Peng Y, Bogden B, Chen Q, Neuhausen S, Shattuck-Eidens D, Godwin AK, Daly M, Radford DM, Sedlacek S, Rommens J, Simard J, Garber J, Merajver S and Weber BL (1996) *BRCA2* germline mutations in male breast cancer cases and breast cancer families. *Nature Genet* **13**: 123–125

Easton DF, Bishop DT, Ford D and Crockford GP. The Breast Cancer Linkage Consortium. (1993) Genetic linkage analysis in familial breast and ovarian cancer: results from 214 families. *Am J Hum Genet* **52**: 678–701

Foster KA, Harrington P, Kerr J, Russell P, DiCioccio RA, Scott IV, Jacobs I, Chenevix-Trench G, Ponder BA and Gayther SA (1996) Somatic and germline mutations of the *BRCA2* gene in sporadic ovarian cancer. *Cancer Res* **56**: 3622–3625

Friedman LS, Gayther SA, Kurosaki T, Gordon D, Noble B, Casey G, Ponder BAJ, Anton-Culver H (1997) Mutation analysis of BRCA1 and BRCA2 in a male breast cancer population. *Am J Hum Genet* **60**: 313–319

Gayther SA, Mangion J, Russell P, Seal S, Barfoot R, Ponder BAJ, Stratton MR, Easton D (1997) Variation of risks of breast and ovarian cancer associated with different germline mutations of the *BRCA2* gene. *Nature Genet* **15**: 103–105

Goggins M, Schutte M, Lu J, Moskaluk CA, Weinstein CL, Petersen GM, Yeo CJ, Jackson CE, Lynch HT, Hruban RH and Kern SE (1996) Germline *BRCA2* gene mutations in patients with apparently sporadic pancreatic carcinomas. *Cancer Res* **56**: 5360–5364

Lancaster JM, Wooster R, Mangion J, Phelan CM, Cochran C, Gumbs C, Seal S, Barfoot R, Collins N, Bignell G, Patel S, Hamoudi R, LC, Wiseman RW, Berchuck A, Iglehart JD, Marks JR, Ashworth A, Stratton MR and Futreal PA (1996) *BRCA2* mutations in primary breast and ovarian cancers. *Nature Genet* **13**: 238–240

© Cancer Research Campaign 1997

Miki Y, Swensen J, Shattuck-Eidens D, Futreal PA, Harshman K, Tavtigian S, Liu Q, Cochran C, Bennett LM, Ding W, Bell R, Rosenthal J, Hussey C, Tran T, McClure M, Frye C, Hattier T, Phelps R, Haugen-Strano A, Katcher H, Yakumo K, Gholami Z, Shaffer D, Stone S, Bayer S, Wray C, Bogden R, Dayananth P, Ward J, Tonin P, Narod S, Bristow PK, Norris FH, Helvering L, Morrison P, Rosteck P, Lai M, Barrett JC, Lewis C, Neuhausen S, Cannon-Albright L, Golgar D, Wiseman R, Kamb A and Skolnick MH (1994) A strong candidate for the breast and ovarian cancer susceptibility gene *BRCA1*. *Science* **266**: 66–71

Miki Y, Katagiri T, Kasumi F, Yoshimoto T and Nakamura Y (1996) Mutation analysis in the *BRCA2* gene in primary breast cancers. *Nature Genet* **13**: 245–247

Neuhausen S, Gilewski T, Norton L, Tran T, McGuire P, Swensen J, Hampel H, Borgen P, Brown K, Skolnick M, Shattuck-Eidens D, Jhanwar S, Goldgar D and Offit K (1996) Recurrent *BRCA2* 6174delT mutations in Ashkenazi Jewish women affected by breast cancer. *Nature Genet* **13**: 126–128

Phelan CM, Lancaster JM, Tonin P, Gumbs C, Cochran C, Carter R, Ghadirian P, Perret C, Moslehi R, Dion F, Faucher MC, Dole K, Karimi S, Foulkes W, Lounis H, Warner E, Goss P, Anderson D, Larsson C, Narod SA and Futreal PA (1996) Mutation analysis of the *BRCA2* gene in 49 site-specific breast cancer families. *Nature Genet* **13**: 120–122

Ravnik-Glavac M, Glavac D and Dean M (1994) Sensitivity of single - strand conformation polymorphism and heteroduplex method for mutation detection in the cystic fibrosis gene. *Hum Mol Genet* **3**: 801–807

Sheffield VC, Beck JS, Kwitek AE, Sandstrom DW and Stone EM (1993) The sensitivity of single-strand conformation polymorphism analysis for the detection of single base substitutions. *Genomics* **16**: 325–332

Stratton MR, Ford D, Neuhasen S, Seal S, Wooster R, Friedman LS, King MC, Egilsson V, Devilee P, McManus R, Daly PA, Smyth E, Ponder BAJ, Peto J, Cannon-Albright L, Easton DF and Golgar DE (1994) Familial male breast cancer is not linked to the *BRCA1* locus on chromosome 17q. *Nature Genet* **7**: 103–107

Struewing JP, Brody LC, Erdos MR, Kase RG, Giambarresi TR, Smith SA, Collins FS and Tucker MA (1995*a*) Detection of eight *BRCA1* mutations in 10 breast/ovarian cancer families, including 1 family with male breast cancer. *Am J Hum Genet* **57**: 1–7

Struewing JP, Abeliovich D, Peretz T, Avishai N, Kaback MM, Collins FS, Brody LC (1995*b*) The carrier frequency of the *BRCA1* 185delAG mutation is approximately 1 percent in Ashkenazi Jewish individuals. *Nature Genet* **11**: 198–200

Takahashi H, Chiu HC, Bandera CA, Behbakht K, Liu PC, Couch FJ, Weber BL, LiVolsi VA, Furusato M, Rebane BA, Cardonick A, Benjamin I, Morgan MA, King SA, Mikuta JJ, Rubin SC and Boyd J (1996) Mutations of the *BRCA2* gene in ovarian carcinomas. *Cancer Res* **56**: 2738–2741

Tavtigian SV, Simard J, Rommens J, Couch F, Shattuck-Eidens D, Neuhausen S, Merajver S, T.S., Offit K, Stoppa-Lyonnet D, Belanger C, Bell R, Berry S, Bogden R, Chen Q, Davis T, Dumont M, Frye C, Hattier T, Jammulapati S, Janecki T, Jiang P, Kehrer R, Leblanc JF, McArthur-Morrison J, Nguyen K, Peng Y, Samson C, Schroeder M, Snyder SC, Steele L, Stringfellow M, Stroup C, Swedlund B, Swensen J, Teng D, Thomas A, Tran T, Tranchant M, Weaver-Feldhaus J, Wong AKC, Shizuya H, Eyfjord JE, Cannon-Albright L, Labrie F, Skolnick MH, Weber B, Kamb A and Golgar DE (1996) The complete *BRCA2* gene and mutations in chromosome 13q-linked kindreds. *Nature Genet* **12**: 333–337

Teng DH, Bogden R, Mitchell J, Baumgard M, Bell R, Berry S, Davis T, Ha PC, Kehrer R, Jammulapati S, Chen Q, Offit K, Skolnick MH, Tavtigian SV, Jhanwar S, Swedlund B, Wong AK and Kamb A (1996) Low incidence of *BRCA2* mutations in breast carcinoma and other cancers. *Nature Genet* **13**: 241–244

The Breast Cancer Information Core http://www.nhgri.nih.gov/Intramural_research/Lab_transfer/Bic/

Thorlacius S, Olafsdottir G, Tryggvadottir L, Neuhausen S, Jonasson JG, Tavtigian SV, Tulinius H, Ogmundsdottir HM and Eyfjord JE (1996) A single *BRCA2* mutation in male and female breast cancer families from Iceland with varied cancer phenotypes. *Nature Genet* **13**: 117–119

Weber BH, Brohm M, Stec I, Backe J and Caffier H (1996) A somatic truncating mutation in *BRCA2* in a sporadic breast tumor. *Am J Hum Genet* **59**: 962–964

Wooster R, Bignell G, Lancaster J, Swift S, Seal S, Mangion J, Collins N, Gregory S, Gumbs C, Micklem G, Barfoot R, Hamoudi R, Patel S, Rice C, Biggs P, Hashim Y, Smith A, Connor F, Arason A, Gudmundsson J, Ficenec D, Kelsell D, Ford D, Tonin P, Bishop DT, Spurr NK, Ponder BAJ, Eeles R, Peto J, Devilee P, Cornelisse C, Lynch H, Narod S, Lenoir G, Egilsson V, Barkadottir RB, Easton DF, Bentley DR, Futreal PA, Ashworth A and Stratton MR (1995) Identification of the breast cancer susceptibility gene *BRCA2*. *Nature* **378**: 789–792

Wooster R, Neuhausen SL, Mangion J, Quirk Y, Ford D, Collins N, Nguyen K, Seal S, Tran T, Averill D, Fields P, Marshall G, Narod S, Lenoir GM, Lynch H, Feunteun J, Devilee P, Cornelisse CJ, Menko FH, Daly PA, Ormiston W, McManus R, Pye C, Lewis CM, Cannon-Albright LA, Peto J, Ponder BAJ, Skolnick MH, Easton DF, Golgar DE and Stratton MR (1994) Localization of a breast cancer susceptibility gene *BRCA2* to chromosome 13q12–13. *Science* **265**: 2088–2090

© Cancer Research Campaign 1997